# Effects of pulsed electromagnetic field therapy on outcomes associated with osteoarthritis: A systematic review of systematic reviews

**DOI:** 10.1007/s00508-022-02020-3

**Published:** 2022-04-01

**Authors:** Lovro Markovic, Barbara Wagner, Richard Crevenna

**Affiliations:** Department of Physical Medicine, Rehabilitation and Occupational Medicine, Medical University of Vienna, Waehringer Guertel 18–20, 1090 Vienna, Austria

**Keywords:** PEMF, Magnetic field therapy, Arthrosis, Umbrella review

## Abstract

**Background:**

Osteoarthritis (OA) is a chronic degenerative disease of multiple joints with a rising prevalence. Pulsed electromagnetic field (PEMF) therapy may provide a cost-effective, noninvasive, and safe therapeutic modality with growing popularity and use in physical medicine and rehabilitation. The purpose of this study was to synthesize the current knowledge on the use of PEMF in OA.

**Methods:**

A systematic review of systematic reviews was performed. The PubMed, Embase, PEDro and Web of Science databases were searched based on a predetermined protocol.

**Results:**

Overall, 69 studies were identified. After removing the duplicates and then screening title, abstract and full text, 10 studies were included in the final analysis. All studies focused on knee OA, and four studies also reported on cervical, two on hand, and one on ankle OA. In terms of the level of evidence and bias, most studies were of low or medium quality. Most concurrence was observed for pain reduction, with other endpoints such as stiffness or physical function showing a greater variability in outcomes.

**Conclusion:**

The PEMF therapy appears to be effective in the short term to relieve pain and improve function in patients with OA. The existing studies used very heterogeneous treatment schemes, mostly with low sample sizes and suboptimal study designs, from which no sufficient proof of efficacy can be derived. A catalogue of measures to improve the quality of future studies has been drawn up.

## Introduction

Osteoarthritis (OA) is a degenerative disease that affects one or more joints and is associated with inflammatory processes in the synovium, loss of cartilage, and alterations of bone structure. An OA can manifest clinically as pain, swelling, deformity, instability, or impaired function in the affected joints. Typical localizations include knee, hip, hand, as well as lumbar and cervical joints [1]. The prevalence of OA is expected to increase in the coming decades due to the aging general population. Globally, the prevalence of knee OA in people aged 15 years and over is around 16%, while the prevalence in people over 40 years of age is much higher at around 22.9%. The pooled global incidence is 203 per 10,000 person-years in those over the age of 20 years, with females and people with obesity being more likely to be affected. Knee OA is the most prevalent form of OA accounting for 75% of the worldwide OA burden [2]. In addition to invasive, operative interventions, a multitude of conservative treatment options are available, especially in the field of physical medicine, including but not limited to physiotherapy, transcutaneous electrical nerve stimulation (TENS), acupuncture, local heat and cold application, as well as pharmacological analgesia, e.g. with non-steroidal anti-inflammatory drugs (NSAID) [3].

Pulsed electromagnetic field (PEMF) therapy is an emerging modality for the treatment of musculoskeletal disorders with a wide range of indications for use and has been approved by the American Food and Drug Administration (FDA) [4]. The PEMF involves time-varying magnetic fields that are generated by strong electrical currents passing through a coil. The frequency, intensity, and shape (i.e. shape of intensity change over time) of these magnetic pulses can be determined and manipulated by physicians [5]. Some of the key advantages of PEMF are the high tolerability due to low side effects, its non-invasive nature and the relatively simple therapeutic applicability. Regarding clinical use, PEMF can be effective in relieving pain and improving functionality in patients with OA, as well as accelerating wound healing, reducing inflammation and treating soft tissue injuries [6]. Although several randomized controlled trials (RCT) have been conducted over the past few decades, there is no consensus or guidelines to help physicians tailor the treatment regimen to their patients, particularly in terms of duration, frequency, and intensity of PEMF therapy sessions.

The evidence for the use of PEMF in patients diagnosed with OA is sparse because the quality, amount, and conclusions of RCTs, as well as systematic reviews do not show conclusive results or the conclusions are based on low level clinical evidence. The aim of the present paper is to provide an overview of application modalities and of the effectiveness of PEMF therapy in patients with OA, to summarize the current state of knowledge and to provide a catalogue of measures to improve the quality of future studies.

## Methods

This systematic review of systematic reviews was conducted based on a preapproved protocol and on the guidelines recommended by the Preferred Reporting Items for Systematic Reviews and Meta-Analyses (PRISMA) statement [7]. The review protocol was not registered.

### Search strategy

The databases PubMed, EMBASE, PEDro, and Web of Science were searched from inception up to 1 July 2021, using a combination of the following terms: “magnetic* field* therap*”, “puls* electromagnetic* field* therap*”, “low* field* magnetic* stimulation*”, “*PEMF”, “*LFMS”, and “osteoarthrit*”, with filters set to only include systematic reviews and/or meta-analyses.

### Inclusion criteria

The inclusion criteria for the studies included in this analysis followed the PICO (population, intervention, control, and outcomes) model:

- Population: patients with OA of one or multiple joints who underwent PEMF therapy alone or in combination with other therapeutic modalities.
- Intervention: studies reporting on the influence of PEMF alone or in combination with other modalities.
- Outcome: studies reporting on the influence of PEMF or any outcome associated with OA.
- Study designs including systematic reviews and meta-analyses of RCTs.

### Exclusion criteria

Studies were excluded for the following reasons:

- Design other than a systematic review (narrative reviews).
- Unavailability of data to be extracted, in this case the corresponding author has been contacted. If no information was available from the corresponding author, the study was excluded.
- Systematic reviews of observational studies.
- Systematic reviews of non-clinical studies or animal model studies.
- Full text articles in a language other than English or German.

### Study selection

Two independent reviewers (LM and BW) conducted a title and subsequent abstract screening. If the inclusion criteria were met, or if further information was needed to determine whether the inclusion criteria were fulfilled, studies were evaluated in full text form. Disagreements between the two reviewers were resolved by discussion and, if necessary, through a third independent reviewer (RC).

### Data extraction and critical appraisal

An extraction plan was created based on the consensus of the authors. Data were tabulated and a narrative synthesis was carried out. The following categories are included in Table 1: name of the first author and year of publication; databases; number and type of studies included; participants: sex, mean age, diagnosis, description and duration of the intervention; control condition; anatomical site of PEMF application; quality assessment tool and its outcomes; outcomes and outcome measures; general conclusion and limitations.

**Table 1:** Overview of included studies

Author and year of publication	Databases	Included studies	Participants; % female; Mean age	Interventions	Control	Duration of intervention and follow up	Magnetic field parameters	Anatomical site	Quality assessment outcome (tool)	Outcome and outcome measures	General conclusion
Yang et al. (2020) [8]	Cochrane Central Register of Controlled Trials, PubMed, CINAHL, Embase, PEDro	Overall 16 studies included. Studies in English reporting on studies in adults with OA who received PEMF as primary treatment and reported on pain, stiffness, physical function and QOL	Adults with OA with a total population *N* = 1078 (554 treatment vs 524 controls). % female: NR. Mean age: 59.5 years	PEMF alone, PEMF with routine PT	Sham PEMF, routine PT with sham PEMF, no treatment and medicine (analgesia when needed)	Duration of intervention (range): 10–36 days; Follow up (range): 10–84 days	Frequency 0.1 Hz‑6.8 MHz; intensity varying from 10–80 Gauss; 7.8 × 10–8 to 1.5 × 10–7; 2–30mT, 40–105 mcT	Knee (*n* = 14); ankle (*n* = 1); hand (*n* = 2); cervical spine (*n* = 2)	Only 2 studies low risk of bias, other studies 14 unclear, 2 high risk (Cochrane, GRADE)	Pain (*n* = 15): VAS or WOMAC pain subscale; Stiffness (*n* = 7): WOMAC stiffness subscale; Physical function (*n* = 8): Lequesne index or WOMAC function subscale; QOL (*n* = 3): EuroQoL or SF-36 scale	PEMF was beneficial in pain reduction regardless of treatment duration, field intensity or frequency. Significant improvements were also seen in stiffness and physical function, albeit for the latter only for duration between 4 and 6 weeks. No differences were seen for QOL. Using MCID results suggest effects on pain reduction are clinically relevant
Viganò et al. (2020) [9]	MEDLINE, Embase, Web of Science, Cochrane Database	Overall 13 studies included. Studies in English reporting on patients with knee OA treated with PEMF that reported pain (VAS) and disability/activity (WOMAC scale)	Adults with knee OA with a total population *N* = 914 (472 treatment vs 442 controls). % female: NR Mean age: NR	PEMF alone, PEMF with PT, SW, TENS and PEMF with ESWT and HP	9 placebo with inactive PEMF device, 4 different combination of other therapeutic modalities (PT, TENS, SW)	Duration of intervention (range): 14–42 days; with some studies reporting 1–30 sessions range between 30 min to 1 h duration. Follow up (range): 0–24 weeks	Frequency between 1–3000 Hz; intensity 3.4 mcT–105 mT, 0.5–30 Gauss, 34 V/m–100 V/cm	Knee	3 studies low risk, 5 studies unclear, 5 studies high risk (Cochrane)	Pain (*n* = 13): VAS, disability associated with knee OA (*n* = 6): WOMAC index	PEMF provided significant pain reduction and WOMAC disability score in knee OA patients in placebo controlled trials. Subgroup analyses showed no difference between PEMF and other theraputic modalities. PEMF is a safe therapeutic option but improvements are similar to other therapeutic modalities (PT, TENS, hyperthermia and ultrasound)
Chen et al. (2019) [10]	PubMed, Embase, Web of Science, Cochrane Library	Overall 8 studies included. Studies in English comparing patients with knee OA treated with PEMF, that reported pain, stiffness, and physical function (WOMAC total, WOMAC stiffness, WOMAC physical function, VAS score)	Adults with OA with a total population *N* = 421 (252 IG vs. 224 CG; there were inconsistency in the results table, authors not available for comment). % female: NR. Mean age: Treatment group range: 55.5–69.2 years; placebo group range: 55.5–67.0 years	PEMF alone	PEMF vs placebo	Duration of intervention (range): 18–45 days; from 5 min 2x/day up to 12 h/day; 1 study reported 18 sessions. Follow-up not reported	Frequency: 1 Hz—6.8 MHz; intensity from 40 mcT to 105mT; one study reporting 34 ± 8 V/m and one 10 mV/cm	Knee	All studies low or moderate risk of bias (Cochrane)	Pain: VAS (*n* = 3), Pain + function: WOMAC (*n* = 4); VAS + WOMAC (*n* = 1)	PEMF had a statistically significant positive effect on physical function as measured by the WOMAC physical function score, compared to placebo. There was no improvement in stiffness or pain. PEMF may be a useful and cost-effective addition to non-interventional treatment of knee OA
Wu et al. (2018) [11]	PubMed, Embase, Web of Science, Cochrane Library	Overall 12 studies included: 10 knee OA, 2 cervical OA, 1 hand OA	Knee *N* = 634; Cervical *N* = 115; Hand *N* = 50; % female: Knee: 8–87.9% (PEMF), 11.5–88.2% (placebo); Cervical: 28.6–64.7% (PEMF), 30.8–66.7% (placebo). Mean age: Knee: 57.7–68.6 (placebo), 52.1–69.2 (PEMF); Cervical: 43.2–61.2 (PEMF), 42.1–67.4 (placebo)	Knee: 7x PEMF, 1x PEMF + hot pack + TENS, 1x PEMS + standard; Cervical: 1x PEMF, 1x PEMS + regimen; Hand: PEMF + AROM + resistive exercise	Placebo	Knee: 3–6 weeks, 1 × 20 sessions. Cervical: 3–5 weeks. Hand: 10 days. Dailty time between 10 min × 3 times a day and a minimum of 12 h	NR	Knee, cervical spine, hand	1 study low quality (Cochrane)	Pain (unclear which scale, change from baseline), physical function (WOMAC, SF-36 social function score, SF-36 global score, or physician global assessment score), adverse events	PEMF proved beneficial for pain reduction and function improvement for knee and hand OA, but not for cervical OA. Overall, a treatment duration of less than 30 mins may be more effective in reducing pain and improving function
We et al. (2013) [12]	MEDLINE, Scopus, Cochrane Central Register of Controlled Trials	Overall 14 studies included with knee OA patients; all were placebo-controlled RCTs	*N* = 930 (482 vs. 448 placebo); % female: 9.8–100%. Mean age: 60.0–73.0 years	PEMF alone	Placebo	Various protocols between 2–6 weeks in duration (one study with 20 session no duration in days indicated); time reported between 6–30 min duration, between 1–8 times a day	Pulse frequency between 1–3000 Hz, intensity: 0.034–69 Gauss, Pule length between 10 mcs and 6 ms	Knee	5 trials low quality (score < 6), 9 high quality (PEDRo, Jadad scale)	Pain and function	No evidence that PEMF was more effective in treating knee pain but was more effective than placebo in improving knee function after 8 weeks following treatment initiation. High quality studies, do however, also suggest effects on pain improvement
Hug, Roosli (2011) [13]	PubMed, Embase, ISI Web of Knowledge, Cochrane Library	4 PEMF RCTs; 3 on knee OA, 1 trial on cervical OA. All studies were double-blind placebo-controlled RCTs	*N* = 255 for OA (131 IG vs. 124 CG); *n* = 223 for knee OA (114 IG vs 109 CG) and *n* = 32 for cervical OA (17 IG vs 15 CG); % female: Knee OA: 49–80%, cervical OA: 66%. Mean age: Knee: 25.2–68, Cervical spine: 42.5 years	Whole-body PEMF, whole-body PEMF + intraarticular steroid injections	Placebo	3–6 weeks (16–30 min/day), 1x only a single session	Intensity from 3.4–105 mcT, frequency from 0.1–3000 Hz (not individually reported)	Knee, cervical spine	NR	Knee: VAS-Score, Lequesne index, KSS, WOMAC OA index and ist subscores, sensory and pain thresholds. Cervical: pain, NPDS, ROM, cervical muscle spasm	Evidence for an effect of whole-body PEMF is insufficient and can thus not be recommended. No short-term side-effects, long-term effects were not examined
Vavken et al. (2009) [14]	PubMed, EMBASE, Cochrane Controlled Trials Register	9 RCTs, comparison of PEMF with placebo	Adults with knee OA (*N* = 483); *n* = 239 (IG), *n* = 244 (CG); % female: 35–91% (IG), 20–72% (CG). Mean age: 58.1–72.7 (IG), 58.3–73.3 (CG) years	PEMF alone (pulsed short wave and classical PEMF)	Placebo	Duration (range): 2–8 weeks; from 15 min 3x/week up to 2 h/day; 1 study reported 18 sessions. Follow up: 4–12 weeks	1 Hz—27 MHz, 3.4 mcT—2.5 mT	Knee	Jadad 5/5 (Jadad)	Pain (*n* = 9): VAS, stiffness (*n* = 4), activities of daily living (*n* = 5), clinical scores (*n* = 4, WOMAC Index, Arthritis Impact Measurement Scale AIMS), end-points abstracted for a time-point as close as possible to 6 weeks of follow-up	No significant effects were reported for pain or stiffness, however there was evidence to support improvements in clinical scores in patients with knee OA. PEMF could be considered as adjuvant therapy modality for knee OA
Bjordal et al. (2007) [15]	Medline, Embase, Cochrane Controlled Trials Register for RCTs, CINAHL, Database of Abstracts of Reviews of Effectiveness, International Network of Agencies for Health Technology Assessment database, PEDro, National Guideline Clearinghouse, PRODIGY Guidance, NICE, hand search	7 PEMF RCTs, placebo-controlled, knee OA verified by clinical examination according to ACR criteria and/or X‑ray with pain > 3 months	*N* = 487; Adults with clinical and radiological confirmation of knee OA; *n* = 255 (IG), *n* = 232 (CG); % female: NR. Mean age: 64.2 years	PEMF (short wave therapy (*n* = 1) and other PEMF (*n* = 6))	Placebo	Short wave therapy: duration NR, 3x/week over 2 weeks; PEMF: 0.5–2 h/day over 6 weeks or 0.5 h 3–5x/week total 18 sessions; one study reported only the performance of 8 sessions without further information	Short wave therapy: 400 Hz, treatment dose 20 k; PEMF: 1–3000 Hz, intensities: 3 × 10–^7^Gauss, < 0.5–15 Gauss, 10 mV, 40 mT	Knee	Jadad mean [range] (all studies): 3.8 [1–5], Jadad mean [range] PEMF studies: 4.4 [3–5]	Pain reduction (VAS pain scale, WOMAC Pain Scale) during the first 4 weeks after initiation of treatment and follow up at 1–12 weeks after end of treatment; global health status 1–12 weeks after end of treatment	Data overall scarce, however some evidence that PEMF offered small improvement in pain outcomes after 4 weeks of therapy, with conflicting results based on different time points. Therefore, it is difficult to provide conclusions on the duration of pain reduction. On patient in one study reported increased pain during treatment and withdrew
McCarthy et al. (2006) [16]	MEDLINE, AMED, EMBASE, HealthSTAR, CINAHL, PEDro, SPORTDiscus, Cochrane Controlled Trials Register (CCTR)	5 RCTs comparing PEMF with placebo; studies using non-validated outcome measures were excluded	Adults with clinical and radiological confirmation of knee OA; *N* = 276; *n* = 138 (IG), *n* = 138 (CG); % female: NR. Mean age: NR	PEMF alone; low frequency PEMF (*n* = 2), “pulsed short wave” high frequency PEMF (*n* = 3)	Placebo	Duration (range): 2–6 weeks; treatment duration: 3–5 h/week (low frequency); high frequency PEMF: NR; follow up period: NR	Low frequency PEMF: 3–50 Hz, high frequency PEMF: NR; intensity: NR	Knee	Jadad 3–5/5	Pain (*n* = 5, VAS Pain Scale, WOMAC Pain Scale), functional disability (*n* = 4, WOMAC Physical Function Scale, AIMS)	Low level of evidence that PEMF provides a substantial contribution to the management of knee OA, with no effects on pain improvement. Some trends point to low frequency and high duration of treatment as effective in improvement of the WOMAC function score
Li et al. (2013) [17]	The Cochrane Central Register of Controlled Trials (CENTRAL), PreMEDLINE, MEDLINE, CINAHL, PEDro, handsearch	9 RCTs, placebo-controlled, treatment duration ≥4 weeks; no language restrictions	Adults with clinical and/or radiological diagnosis of OA (ACR criteria), trials with previous surgical treatment of OA excluded; *N* = 636, *n* = 327 (IG), *n* = 309(CG); % female: NR. Mean age: NR	Electromagnetic field interventions (PEMF (*n* = 6) and pulsed electrical stimulation (*n* = 3))	Placebo	PEMF: 0.3–1.5 h/day or 0.5 h 3–5x/week for 4–6 weeks; Pulsed electrical stimulation: 6–14 h/day for 4–26 weeks	PEMF: 1 Hz—6.8 MHz, intensities: 40 mcT—105 mT, 34 ± 8 V/m, 10 mV/cm pulsed electrical stimulation: 100 Hz	Knee (*n* = 7), OA in general (*n* = 1), knee, cervical spine (*n* = 1)	Inadequate reporting of study design and conduct (*n* = 9), high risk of bias for incomplete outcome data (*n* = 3). Overall risk of bias was low for the other domains across the 9 studies	Pain (*n* = 6, VAS Pain Scale), physical function (*n* = 3, WOMAC Physical Function Scale), health-related quality of life (*n* = 2, SF-36), radiographic joint structure changes (*n* = 1, bone scintigraphic examination), number of patients experiencing adverse events (*n* = 4), number of patients who withdrew because of adverse events (*n* = 1), number of patients experiencing any serious adverse event	Some evidence to support a moderate benefit for OA patients in terms of pain reduction, no conclusive evidence to support improvements in physical functioning or to general health and well-being

*ACR* American College of Rheumatology, *AIMS* Arthritis Impact Measurement Scales, *CG* control group, *ESWT* extracorporeal shock wave therapy, *HP* hot pack, *IG* intervention group, *KSS* Knee Society Score, *mcT* microtesla, *mT* militesla, *MCID* minimal clinically important difference, *NPDS* Neck Pain and Disability Scale, *NR* not reported, *OA* osteoarthritis, *PEMF* pulsed electromagnetic fields, *PT* physical therapy, *QOL* quality of life, *RCT* randomized controlled trial, *SW* ultrasound therapy, *TENS* transcutaneous electrical nerve stimulation, *VAS* Visual Analogue Scale, *WOMAC* Western Ontario and McMaster Universities Osteoarthritis Index

## Results

**Fig. 1:**
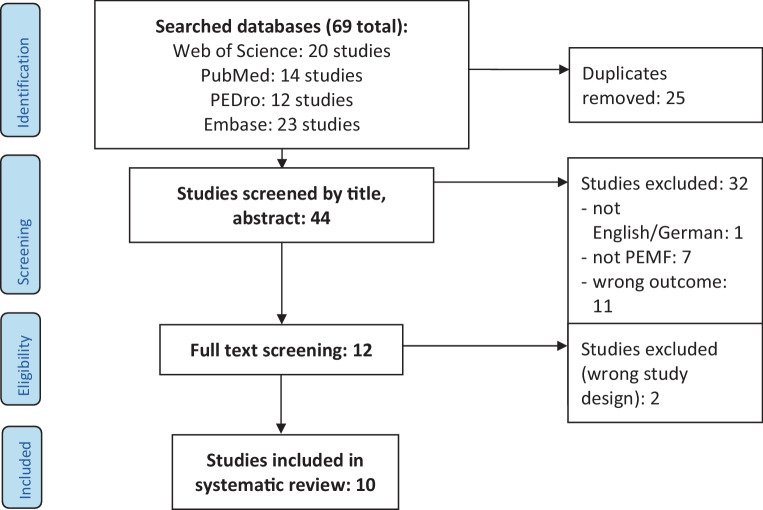
Flowchart of systematic literature search and selection according to PRISMA guidelines

Our systematic review includes 10 systematic reviews that focus on the effect of PEMF on a variety of outcomes in patients with OA, as presented in Table 1. An overview of the literature search and selection process are presented in Fig. 1.

In terms of localization, all systematic reviews include results on knee OA [8–17], with four reviews additionally including cervical spine [8, 11, 13, 17], two studies reporting on hand OA [8, 11], and one on ankle OA [8]. All included reviews report on the outcomes of individual studies in adults, with a mean age range between 25 and 73 years.

All included systematic reviews reported outcomes on disability or physical function and used the Western Ontario and McMaster Universities Osteoarthritis Index (WOMAC) [18] as a measurement for physical function or disability. One study additionally reports on activities of daily living. Out of 10 studies, 5 report positive outcomes associated with the application of PEMF in patients with OA [8–11, 17] and 1 study reports no statistically significant effect of PEMF ([14]; Table 1).

Pain was assessed as an outcome in all of the systematic reviews, with all reviews reporting results of the visual analogue scale and one not reporting which scale was used. In total, five studies report that PEMF had significant effects on pain reduction in patients with OA [8, 9, 11, 15, 17].

Joint stiffness and quality of life were assessed in two reviews reported here [8, 14]. Joint stiffness was assessed using the WOMAC stiffness subscale and quality of life using the SF-36 and EuroQol scales [8, 11]. Overall, reviews report no positive effects on quality of life in patients using PEMF and only one review found significant improvements in joint stiffness [8].

Treatment protocols were very heterogeneous. One review limited the devices studied to full-body mats [12]. The PEMF field intensity varied between 3.4 mcT [9, 13, 14] and 105 mT [8–10, 13, 17], with most of the studies being carried out in the millitesla range. Several studies used other units to state magnetic flux density such as Gauss, V/cm or V/m. Treatment frequencies ranged from 0.1 Hz [8, 13] to 27 MHz [14]. Two trials did not provide any information on the field intensity and frequency that was applied [11, 16]. Waveform, if indicated, again was quite different in the respective trials.

Regarding the duration of the intervention, treatments were applied for 6 min [8] to 12 h a day [8, 9, 11], daily [10] to three times a week [10] and over a time period of ten [8] to 45 [9] days.

## Discussion

Since previous systematic reviews and meta-analyses of RCTs often reported contradicting evidence regarding the effectiveness of PEMF in patients with OA, we aimed to provide a comprehensive literature synthesis through a systematic review of systematic reviews in order to gain more insight into the current state of research. Overall, our results show that there is some degree of congruency between studies in the effectiveness of this type of therapy in terms of physical functioning or reduction of disability and pain; however, the discrepancies on the reported outcomes on effectiveness among studies are large and do not allow unequivocal conclusions on the effectiveness of PEMF. The main results and characteristics of the included studies are presented in Table 1.

Previous studies [11, 16] have reported conflicting results on physical function outcomes; however, the majority of the systematic reviews included in our review suggest a positive effect of PEMF. Some studies have reported the potential mechanisms by which PEMF can relieve pain, emphasizing its role in diminishing proinflammatory cytokines, as well as increasing chondrocyte proliferation and extracellular matrix production. Reducing pain may also be one of the reasons for improvement in physical functioning and reducing the level of disability. When comparing studies and interpreting the results, the specific physical parameters of electromagnetic devices must be taken into account [19]. Necessary details that characterize an electromagnetic device, such as the type of the field, the intensity of the induction, frequency, rise and decline of the pulse rate, pulse shape and vector or exposure time, are rare information and vary between different treatment protocols. Therefore, comparisons between existing studies and qualified ratings are often difficult [19]. This was confirmed through the results of our analysis. Although most studies focused on knee localization and used the WOMAC scale, the differences between intervention protocols (duration, intensity, and frequencies of the magnetic field) preclude the possibilities of further meaningful comparisons. Given these differences, a meta-analysis was not possible either. Special features of the various devices only allow a comparison with respect to comparable physical parameters [19].

Moreover, there are few studies using high intensity magnetic fields that are most likely to produce a physiological response.

There are no guidelines or a clear professional consensus on the use of PEMF in the treatment of OA. Reporting the duration of the exposure to the electromagnetic field is particularly important, as a recent study on mesenchymal stem cell differentiation pointed out that the expression of chondrogenic markers was greatest with treatments lasting between 5 and 20 min [20]. There is evidence to suggest that PEMF can induce cellular signaling transduction within 5–10 min, while signaling is largely depleted after 30 min [21–23].

While most studies reported outcomes on knee OA, those that reported on cervical OA mostly found minor effects for this patient population. This may be due to the neural and vascular structures that may compress the cervical canal and lead to a number of symptoms including numbness of the limbs, falls, and pain in the nerve root of the upper limb [24, 25]. There is no evidence that PEMF can reduce the formation of osteophytes, which often lead to compression of the nerve root and resulting pain and loss of function [26, 27].

The limitations of our systematic review are mostly related to the individual limitations of the included reviews, which are predominantly due to the small number of participants in the included studies and the high heterogeneity of the interventions and outcomes. The main limitation of this systematic review of systematic reviews is the small number of studies that could be included. Moreover, we only included studies that were published in English and German.

## Conclusion and implications for future research

The results of our review suggest that the use of PEMF is a safe and noninvasive therapy option for patients with OA that can lead to improvements in pain and physical function.

Future studies should aim to:

- Further improve the quality of future studies, for example by aiming for a more meticulous study design and by ensuring proper blinding and randomization in larger and better defined samples, in order to further improve the quality and level of evidence for the use of PEMF in patients with OA.
- Conduct future trials with homogeneous outcome assessment (to enable future meta-analysis).
- Achieve an international consensus on the uniform reporting of the magnetic flux density of the applied electromagnetic fields, such as microtesla/millitesla or Gauss, in order to be able to better compare study protocols.
- Standardize additional therapeutic modalities, such as physiotherapy, hyperthermia, TENS, or ultrasound if these modalities are used in conjunction with PEMF to enable meaningful comparisons between groups.
- Provide sufficient information on the treatment protocol (e.g. frequency, intensity, waveforms, treatment duration) and on therapy adherence.
- Evaluate the optimal type, frequency, intensity and duration of PEMF interventions in order to develop standardized protocols. It can make sense to homogenize interventions according to the particular physical parameters of the applied electromagnetic fields as well as according to the duration of treatment and treatment indication.
- Evaluate the effect of PEMF on osteoarthritic conditions other than the knee, for example in patients with coxarthrosis
- Continue to evaluate the safety of PEMF interventions (especially when high-intensity protocols are used over a long period of time)
- Evaluate a shorter duration of the electromagnetic fields in RCTs, as there is limited evidence that they affect cellular changes. Similarly, evaluate protocols using high-intensity magnetic fields in the millitesla range that allow sufficient penetration of body tissues as they are likely to produce a stronger physiological response.
